# Intensity modulated radiation therapy versus volumetric intensity modulated arc therapy

**DOI:** 10.1002/jmrs.19

**Published:** 2013-08-22

**Authors:** Suresh Rana

**Affiliations:** Department of Medical Physics, ProCure Proton Therapy CenterOklahoma City, Oklahoma, USA

The advanced developments in external beam radiation therapy (EBRT) over the past few decades have improved dose conformity to the target while minimizing dose to the surrounding organs at risk (OAR). Intensity modulated radiation therapy (IMRT) and volumetric modulated arc therapy (VMAT) are two commonly used EBRT techniques to treat cancer. In sliding window (SW) or dynamic IMRT, each radiation beam is modulated by continuously moving multileaf collimators (MLC), whereas in step-and-shoot (SS) or static IMRT, the MLC divide each radiation beam into a set of smaller segments of differing MLC shape, and the radiation beam is switched off between the segments.^1^,^2^ The modulation of beam intensity within each treatment field leads to construction of conformal dose distributions around the target volume. However, the delivery of a modulated IMRT plan takes longer than the delivery of a nonmodulated three-dimensional (3D) plan due to increased number of monitor units (MU). In contrast, the VMAT can decrease the treatment delivery time as VMAT has more beam entry angles, which likely contributes to the lower number of MU needed compared with the IMRT plan. In the VMAT, one or multiple arcs are used for the treatment, and the delivery technique allows the simultaneous variation in gantry rotation speed, dose rate, and MLC leaf positions.^1^,^2^

The advanced developments in external beam radiation therapy (EBRT) over the past few decades have improved dose conformity to the target while minimizing dose to the surrounding organs at risk (OAR). Intensity modulated radiation therapy (IMRT) and volumetric intensity-modulated arc therapy (VMAT) are two commonly used EBRT techniques to treat cancer. In sliding window (SW) or dynamic IMRT, each radiation beam is modulated by continuously moving multileaf collimators (MLC), whereas in step-and-shoot (SS) or static IMRT, the MLC divide each radiation beam into a set of smaller segments of differing MLC shape, and the radiation beam is switched off between the segments.^1^,^2^ The modulation of beam intensity within each treatment field leads to construction of conformal dose distributions around the target volume. However, the delivery of a modulated IMRT plan takes longer than the delivery of a nonmodulated three-dimensional (3D) plan due to increased number of monitor units (MU). In contrast, the VMAT can decrease the treatment delivery time as VMAT has more beam entry angles, which likely contributes to the lower number of MU needed compared with the IMRT plan. In the VMAT, one or multiple arcs are used for the treatment, and the delivery technique allows the simultaneous variation in gantry rotation speed, dose rate, and MLC leaf positions.^1^,^2^

Recently, there has been increased interest in treating cancer using VMAT. Several authors have done the treatment planning studies comparing IMRT versus VMAT for different tumour sites,^2^^–^9 but the findings from one study are conflicting with those of another study in some cases. For example, current literature comparing VMAT and IMRT for a lung tumour^3^ shows that both techniques could provide comparable target coverage and dose conformity. However, the OAR results in the case of lung tumour are contradictory among different studies. Rao et al. showed that the relative volume of normal lung receiving 20 Gy (V_20_) was higher in the VMAT plans than in the IMRT plans.^3^,^4^ In contrast, Verbakel et al. showed that the VMAT and IMRT plans achieved comparable V_20_ of normal lung.^3^,^5^ The planning studies of prostate cancer have produced inconsistent results too. Yoo et al.^2^,^6^ reported lower doses to the OAR in the IMRT plans than in the VMAT plans, but Ost et al.^2^,^7^ showed that VMAT was better at reducing rectal dose compared to IMRT. Furthermore, the planning techniques within the VMAT have shown inconsistent results as well. For prostate cancer, in comparison to the single-arc technique (SA), Sze et al.^8^ reported that the double-arc technique (DA) produced higher bladder dose, whereas Yoo et al. showed that the DA produced lower doses to the bladder.^2^,^6^ Guckenberger et al.^9^ showed that the DA yielded higher rectal dose, whereas Sze et al. reported lower rectal doses with the DA when compared to the SA.^2^,^8^

The inconsistency in the results among different planning studies may have been due to difference in selection of beam parameters, dose calculation algorithm, plan optimization technique, and delivery technique of the treatment machine. In comparison to the VMAT plan with one arc, the VMAT plan with multiple arcs has more control points that give higher degree of freedom for possible MLC positions. This could result in higher degree of modulation and better plan quality, especially for a complex-shaped target volume. However, a higher degree of modulation generally increases the planning time due to longer plan optimization and dose calculation processes. Thus, treatment planning personnel may be required to make a compromise between planning time and plan quality depending on the physician requirements and available planning resources. The dosimetric results of the OAR can also be affected by the design of the treatment machine as dose to the OAR is dependent on the secondary collimator transmission and scatter radiation of the machine.

Another factor that may affect the quality of the IMRT and VMAT plans is the dose calculation algorithm. Several studies have shown that dose calculation algorithms employed within commercially available treatment planning system (TPS) are not consistent in predicting doses, especially when heterogeneous media are involved along the photon beam path. The difference in beam modelling within dose calculation algorithms may result in different dosimetric results. If the treatment plan includes a small lung tumour, dose calculation algorithms must apply tissue heterogeneity corrections that will account accurately for the electronic disequilibrium effect near the air/tissue interfaces. The International Commission on Radiation Units (ICRU) recommends the dose to be delivered with an error of less than 5%. This implies that necessary accuracy for the dose calculation on treatment plans should be on the order of 2–3%. However, photon dose calculation algorithms could have dose prediction error more than 3% depending on the field size of a photon beam and tissue heterogeneities.^10^,^11^ Thus, the dosimetric results of planning study using one dose calculation algorithm may differ from those of planning studies that used different dose calculation algorithms.

The quality of treatment plan is also dependent on the dose–volume (planning) objectives and planner's familiarity with certain algorithms/interfaces. For example, during the treatment plan optimization in the Eclipse TPS, a planner has the flexibility of selecting dose–volume objectives and weight factor to generate an optimum treatment plan. Additionally, a planner who has worked with IMRT for several years will likely be better in creating IMRT plans than VMAT plans, if the controls and/or optimization parameters are different. Direct comparison between different studies using IMRT and VMAT is also not straightforward because of the differences in prescription dose, planning target volume definitions, and plan optimization algorithms.

There is no doubt that the dosimetric results in the treatment plan play an important role in selecting the treatment technique; however, it is important to note that the quality assurance (QA) result of a patient treatment plan could also impact the selection of IMRT or VMAT technique. The choice of measurement device is also equally important to verify the patient-specific QA. For instance, the MapCHECK 2D diode array is typically used for the IMRT technique, whereas the ArcCHECK 3D diode array is used for the VMAT technique. Sanghangthum et al.^12^ reported that the QA results using ArcCHECK for the VMAT were surprisingly better than those of MapCHECK for the IMRT. In that study, both the VMAT and IMRT plans were generated in the Eclipse TPS, and the authors pointed out that the VMAT in the Eclipse TPS is better than the IMRT for fluence map segmentation.^12^ Although the passing rate of the VMAT QA may be higher than that of the IMRT QA,^12^ the QA results of the VMAT and IMRT cannot be directly compared because of the difference in the delivery technique and measurement devices used to verify the QA plans.

In the recent years, the development of new treatment delivery methods, dose calculation engines, and plan optimization algorithms has led to an increased number of planning comparison studies, which are mainly based on the statistical analysis of dose–volume histogram data. Furthermore, the treatment planning studies typically report the average results of a group of patients, and patient-specific results are usually not discussed. It is important to note that the difference in tumour location in patients with different anatomy may also provide different dosimetric results. As clinical protocols may include the data from different centres with different TPS/algorithm, it is essential to have a database that includes the patient characteristics, treatment planning and optimization parameters, delivery technique, and patient follow-up information. This could provide us some guidelines to compare the treatment plans generated by different planning techniques. However, it may not be possible to completely eliminate the planner bias among different planning studies. Due to dependency of dosimetric quality of IMRT and VMAT on different factors mentioned in this article, the results from the treatment planning studies must be interpreted with caution.
